# Ion Liquid Modified GO Filler to Improve the Performance of Polymer Electrolytes for Li Metal Batteries

**DOI:** 10.3389/fchem.2020.00232

**Published:** 2020-03-31

**Authors:** Zhongliang Hu, Xiaojing Zhang, Jilei Liu, Yirong Zhu

**Affiliations:** Department of Inorganic Nonmetallic Material, College of Metallurgy and Material Engineering, Hunan University of Technology, Zhuzhou, China

**Keywords:** graphene oxide, ionic liquid, polymer electrolyte, Li metal anode, Li metal batteries, rechargeable batteries

## Abstract

Polymer electrolytes for Li metal batteries (LMBs) should be modified to improve their ionic conductivity and stability against the lithium electrode. In this study, graphene oxide (GO) was modified by ion liquid (IL), and the IL modified GO (GO-IL) had been used as a filler for polyethylene oxide (PEO). The obtained solid polymer electrolyte (SPE) is of high ionic conductivity, low crystallinity and excellent stability against the lithium electrode. The PEO/GO-IL was characterized by various techniques, and its structure and performance were analyzed in detail. By addition of 1% GO-IL, the ionic conductivity of the PEO/GO-IL SPE reaches 1.8 × 10^−5^ S cm^−1^ at 25°C, which is 10 times higher than PEO (1.7 × 10^−6^ S cm^−1^), and the current density for stable Li plating/stripping in PEO/GO-IL can be increased to 100 μA cm^−2^ at 60°C. LiFePO_4_/Li cell (using PEO/GO-IL SPE) tests indicated that the initial discharge capacity can reach ~145 mA h g^−1^ and capacity retention can maintain 88% even after 100 cycles at a rate of 0.1C and at 60°C. Our creative work could provide a useful method to develop SPEs with excellent performance, thus accelerating the commercial application of LMBs.

## Introduction

Li metal anodes have a high theoretical capacity (3,860 mA h g^−1^) and extremely low redox potential (~3.04 V vs. standard hydrogen electrodes), so Li metal batteries (LMBs) have been thought as promising secondary power sources for next generation of cutting-edge devices such as electric vehicles, autonomous aircrafts and smart grids (Zhang et al., 2016; Cheng et al., 2017; Wang et al., 2019; Cai et al., 2020; Wu et al., 2020; Zhu et al., 2020; Liao et al., in press). However, Li dendrite growth would happen during Li plating/stripping, probably resulting in short circuit and performance deterioration (Xu et al., 2014; Gao et al., 2019; Yan et al., 2020). In addition, liquid organic electrolytes commonly used in Li ion batteries are flammable and easy-leakage, and hence they would bring more severe safety problem when used in LMBs (Fu et al., 2016). Therefore, solid electrolytes have attracted increasing attention owing to their non-flammability, high safety, and good flexibility (Fan et al., 2018; Zhang et al., 2018).

At present mainly two kinds of solid electrolytes with quite different characteristics have been explored, which can be classified into inorganic electrolytes and polymer electrolytes (Fu et al., 2016). Although inorganic electrolytes have high ionic conductivities (~10^−3^ S cm^−1^) at room temperature, they are stiff and fragile, resulting in their poor processability and huge interfacial resistance between them and Li metal anodes. On the contrary, solid polymer electrolytes (SPEs) containing lithium salts can be easily processed into a membrane owing to their properties of flexibility and stretchability. The commonly investigated polymers as SPEs include poly(ethylene oxide) (PEO) (Ito et al., 1987), polyacrylonitrile (PAN) (Slane and Salomon, 1995), poly(methyl methacrylate) (PMMA) (Appetecchi et al., 1995), and poly(vinylidene fluoride-co-hexafluoropropene) (PVDF-HFP) (Boudin et al., 1999). Among them, PEO has been considered as the most appropriate polymer to prepare SPEs because PEO molecule has rich lone pair electrons in its O atoms, thereby endowing it with strong coordination ability (Mohanta et al., 2018). Unfortunately, the ionic conductivity of PEO is relatively low (~10^−6^ S cm^−1^ at room temperature) (Zhang Q. et al., 2017), and obviously it can not reach the required value (>10^−5^ S cm^−1^) for SPEs of LMBs at ambient and moderate temperature.

Li ions can transport along with the PEO chains, so the ionic conductivity of PEO can be enhanced by various means of lowing its crystallization, including adding plasticizers (Zhang D. et al., 2017), mixing with other co-polymers (Puthirath et al., 2017) and introducing inorganic particles (Zhang J. et al., 2017). The investigation conducted by Cui's group demonstrated that the ion transport in polymers can be effectively ameliorated and their good physicochemical properties are well-maintained by incorporating inorganic fillers into polymers (Lin et al., 2015).

Recently, due to its unique structure and special characteristics, graphene oxide (GO) has been regarded as a promising active nanofiller to improve the performance of SPEs. GO has rich oxygen-containing groups (epoxy, hydroxyl, carboxyl, etc), and these groups can attract Li ions by acid-base interactions, consequently further promoting the dissociation of the lithium salt into free ions. Furthermore, GO can facilitate creating continuous ion channels within polymer electrolytes (Shim et al., 2014). The investigation conducted by Ardebili indicated that addition of 1% GO filler can induce 260% increase in tensile strength, and significantly improve thermo-mechanical stability of the polymer electrolyte (Yuan et al., 2014). However, GO tends to restack at high temperature owing to strong interlayer interaction, thus drastically expediting the capacity fading and greatly lowing the coulombic efficiencies (Yang et al., 2013).

On the other hand, recently the incorporating ionic liquids (ILs) into the polymer electrolytes becomes a popular approach to enhance their conductivity (Shin et al., 2005). Their beneficial properties such as non-volatility, non-flammability, high conductivity and high thermal stability can make them be excellent dopants for polymer electrolytes (Kar et al., 2019). We conceived that the filler of IL modified GO can well-improve the performance of PEO. Firstly, GO can coordinate with oxygen atoms in PEO chains, thereby effectively reducing PEO's crystallization and weakening the interactions between PEO and ions as well as those among various ions (Lee et al., 2015). Secondly, the cations in ILs can also lower the interaction between Li^+^ and PEO, thus promoting the migration of more lithium ions (Long et al., 2016). Finally, ILs can inhibit the aggregation of GO sheets by reacting IL-NH_2_ with GO (Lin et al., 2014). The IL modified filler is expected to not only sharply enhance Li ion migration number but also greatly increase mechanical strength of SPEs.

In this work, 1-(3-aminopropyl)-3-methylimi-dazolium bromide (IL-NH_2_), and graphite oxide were used as precursors to synthesize IL-GO filler, which was adopted to modify PEO. The resultant PEO/GO-IL electrolyte possesses a high ionic conductivity at 25°C (1.8 × 10^−5^ S cm^−1^), can effectively suppress the growth of Li dendrites on Li metal anode, and the LMBs with PEO/GO-IL electrolyte display good performance. The study could provide a methodlogy to solve the dilemma of LMBs by designing novel SPEs with excellent performance.

## Experimental

### Materials

Graphite powders (100 mesh, Nanjin XFNANo), LiFePO_4_ cathode slurry (MTI Co.), Lithium bis(trifluoromethanesulfonyl) imide (LiTFSI, Alfa Aesar), Poly(ethylene oxide) (PEO, Mw=1000000, Sigma), 1-(3-aminopropyl)-3-methylimidazolium bromide (Lanzhou Institute of Chemical Physics), Potassium permanganate (KMnO_4_, Sinopharm), Sulphuric acid (H_2_SO_4_, concentrated, Sinopharm), Hydrogen peroxide (H_2_O_2_, Sinopharm), Potassium hydroxide (KOH, Sinopharm), Sodium nitrate (NaNO_3_, Sinopharm).

### Preparation of Graphite Oxide

Graphite oxide was prepared following the Hummers' method (Hummers and Offeman, 1958). Briefly, 4 g of graphite powder and 4 g of NaNO_3_ were put into a flask, and then 200 mL of concentrated H_2_SO_4_ was added into the flask at 0°C under vigorous stirring. Atfer 24 g of KMnO_4_ was gradually added, the mixture solution was first stirred at 0°C for 2 h and then at 35°C for 2 h. Subsequently 120 mL of distilled water was slowly dropped to cause an increase in temperature to 98°C and this temperature was held for 25 min. After 200 mL of distilled water was again added into the mixture solution, 30 mL H_2_O_2_ was added, ensuring that the residual permanganate and manganese dioxide were reduced to manganese sulfate. Finally, the reaction product was repeatedly washed with distilled water, centrifuged until the supernatant solution became neutral. The graphite oxide was collected, dried and preserved in a desiccator.

### Preparation of GO-IL Filler

IL-GO was synthesized by an epoxide ring-opening reaction between GO and IL-NH_2_ (Yang et al., 2009; Lin et al., 2014). Firstly, 20 mg of IL-NH_2_ and 20 mg of KOH were successively added into 20 mL of graphie oxide (0.5 mg/mL) solution, and the obtained turbid solution had been treated by ultrasonication until it became a homogeneous solution. Subsequently the mixture solution was transferred into a water bath to reflux at 80°C for 24 h. The solid reaction product was centrifuged and washed with ethanol to remove the unreacted IL-NH_2_. Finally 10 g of the solid product and 50 mg of LiTFSI were successively added into 20 mL of distilled water, and the mixture solution reacted at 50°C for 8 h under stirring. After centrifugation and washing with distilled water, the final GO-IL filler was obtained.

### Preparation of Electrode

The cathode slurry (the weight ratio of LiFePO_4_, Super P and PVDF is 8: 1: 1, NMP solvent) was evenly coated on an aluminum foil and then dried at room temperature to form a film. The loading mass of slurry in the film was ensured to be 2.5~3.0 mg cm^−2^, and as-obtained LiFePO_4_ (LFP) material was used as a cathode of LMB for later electrochemical tests.

### Preparation of PEO/GO-IL SPE

The PEO/GO-IL SPE was prepared by solvent casting technique. 10 mg of the IL-GO filler was added into 10 g of absolute acetonitrile. After 2 h ultrasonication and subsequent 12 stirring, the mixture solution become homogeneous. Then, 800 mg of PEO and LiTFSI (the molar ratio of EO and Li is 20: 1) were added into the above solution and stirred for 12 h, resulting in a uniform and brown slurry. The above slurry was spread on a round dish and heated at 45°C overnight to evaporate acetonitrile. Finally, the resultant membrane was peeled off from the dish and dried under vacuum at 45°C for 12 h to obtain the PEO/GO-IL SPE.

### Materials Characterization and Electrochemical Measurements

The powder X-ray diffraction (XRD) patterns were collected using a Rigaku D/max-2500 with Cu Ka radiation. Atomic force microscope (AFM) images were obtained with a Digital Instruments nanoscope IIIa (Multimode, Veeco), operating in tapping mode. Fourier transform infrared (FTIR) spectra were recorded using a FTIR spectrometer (VERTEX 70v spectrometer, Bruker) in the wave number range of 400–4,000 cm^−1^. Scanning electronic microscopy (SEM) images were performed using a Hitachi SU8020 electron microscopy.

The cells were assembled in an argon-filled glove box. The half-cells were charged and discharged with a constant current between 2.5 V and 4.0 V. The charge/discharge cycling studies were performed on a battery test system (LAND CT2001, China). Electrochemical impedance spectra tests (EIS) were carried out in the frequency ranging from 0.1 to 100 KHz in an electrochemical workstation (Autolab). Linear sweep voltammetry (LSV) was conducted in an electrochemical workstation (Interface-1000E, Gamry) using Li/composite/stainless steel cells and the sweeping voltage ranged from 2.6 to 6.0 V with a scanning rate of 10 mV s^−1^.

## Results and Discussions

**Figure 1:**
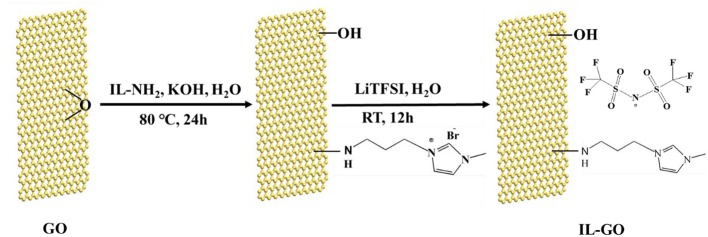
Sketch of the synthesis processes of GO-IL.

The sketch of the synthesis processes of GO-IL filler is shown in Figure 1. The graphite oxide prepared by Hummers' approach can act as a good precursor for GO and it can easily form GO solution under ultrasonication when dispersed in alkaline solution. The GO reacted with IL-NH_2_ by epoxide ring-opening reaction between epoxy groups and -NH_2_ groups in the precursors (Yang et al., 2009), and then reacted with LiTFSI by anion-exchange, forming the GO-IL filler.

**Figure 2:**
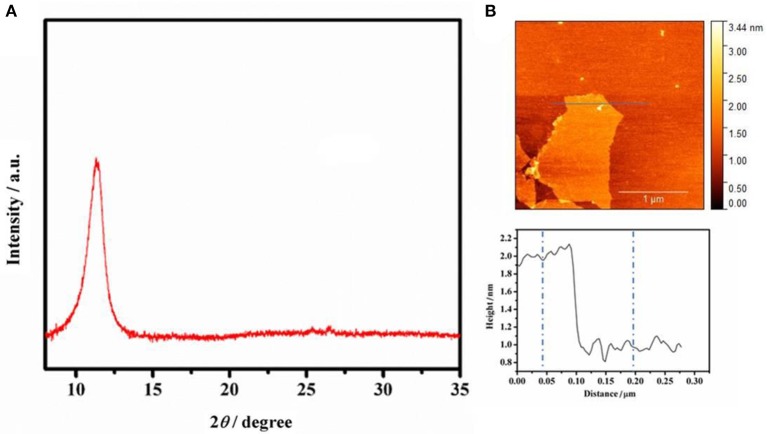
**(A)** XRD spectrum of graphite oxide. **(B)** AFM image of GO (0.25 mg mL^−1^ GO water solution) on freshly cleaved mica surface through drop-casting, and height profiles along the line shown in AFM image.

Figure 2A shows the XRD pattern of graphite oxide, the precursor of GO. A peak at 12.2° can be clearly observed, which can be attributed to the (001) crystal plane of graphite oxide. The XRD characterization demonstrates that graphite oxide has a good crystalline structure. However, when it is dispersed in water or a base solution, it can easily dissociate into single layer of graphene oxide, which was proved by AFM characterization. Figure 2B presents the AFM image and corresponding height profile of GO, and the thickness of GO is ~1.05 nm, indicating that GO is of single layer (Zhu et al., 2010).

**Figure 3:**
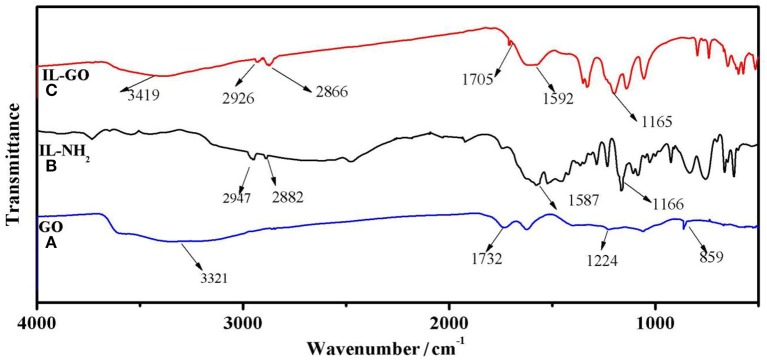
FT-IR spectra of (A) GO,(B) IL-NH_2_, and (C) GO-IL.

Figure 3 displays the FTIR spectra of GO, IL-NH_2_, and IL-GO. For GO, the peak at 1,732 cm^−1^ can be attributed to the stretching vibration of carboxyl groups of GO. The peak at ~859 cm^−1^ comes from epoxy groups, and the broad peak centered at 3,321 cm^−1^ can be ascribed to the stretching vibration of hydroxyl groups of GO. For IL-NH_2_, The peak at ~2,947 and 2,882 cm^−1^ can be assigned to the stretching vibrations of methylene and methyl, respectively. The peak at 1,587 cm^−1^ is due to imidazolium cations. For GO-IL, several new peaks at 2,926, 2,866, and 1,592 cm^−1^ can be observed, inferring that GO has been successfully bonded with IL-NH_2_. In addition, the peak belonging to epoxy groups of GO disappears in GO-IL, further demonstrating GO has successfully reacted with IL-NH_2_.

**Figure 4:**
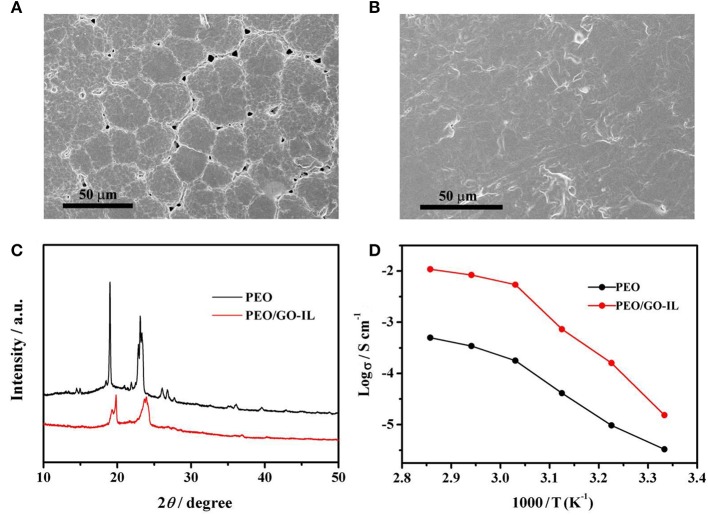
SEM images of **(A)** PEO, **(B)** PEO/GO-IL, **(C)** XRD patterns of PEO and PEO/GO-IL, **(D)** ion conductivity vs. temperature of PEO and PEO/GO-IL.

The AFM image in Figure 2 has fully proved that the GO is of single layer with a small size and the nanostructured GO-IL filler can be evenly distributed in PEO matrix. The SEM images of PEO and PEO/GO-IL are presented in Figure 4. Obviously, there exist very clear grain boundaries in PEO, and in some places the PEO crystals are evenly separated from each other by some pores, indicating that PEO has a good crystalline texture. In contrast, PEO/GO-IL does not exhibit any crystal boundaries (Figure 4B), and instead a smooth and uniform surface is presented, inferring that the crystallinity of PEO has been effectively lowed.

The XRD patterns of PEO and PEO/GO-IL are displayed in Figure 4C. Both samples have peaks at 2θ = 19.2 and 23.3°, which can be ascribed to the characteristic peaks of PEO. However, in contrast to PEO, PEO/GO-IL presents much broader and weaker peaks, indicating that the crystallinity of PEO in PEO/GO-IL has deteriorated greatly. Therefore, the XRD results further proved that the addition of GO/IL fillers can significantly low the crystallinity of PEO, in good agreement with SEM results.

The evolutions of the ionic conductivity with temperature were investigated and the results are presented in Figure 4D. Obviously, PEO/GO-IL (1.8 × 10^−5^ S cm^−1^) has a much higher ionic conductivity than PEO (1.7 × 10^−6^ S cm^−1^) at 25°C, and it can be due to two reasons. Firstly, the addition of GO-IL decreased the crystallinity of PEO, which was beneficial to enhancing the ionic conductivity. Secondly, GO can dissociate lithium salt into free ions and facilitate creating continuous ion channels within polymer electrolytes (Shim et al., 2014). The structure variation of PEO in the electrolyte can be attributed to the addition of GO-IL, in which IL-NH_2_ has reacted with GO by covalent bonding, thereby inhibiting the agglomeration of GO sheets. In this study, GO-IL can promptly increase the ionic conductivity at a low content. Unfortunately, when the content of GO-IL is >1%, the ionic conductivity will no longer improve and even decrease slightly with further increasing the GO-IL content. Hence the optimal content of GO-IL is 1%. The utilization of IL to modify GO via chemical bonding can make good use of the advantages of GO and IL, and it has be proved to be a promising way to enhance the performance of SPEs.

**Figure 5:**
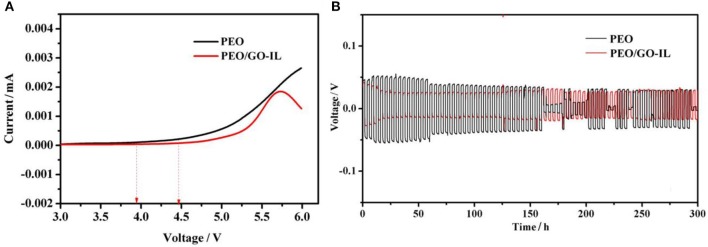
**(A)** Linear sweep voltammetry scans for PEO and PEO/GO-IL. **(B)** Galvanostatic cycles under 0.1 mA cm^−2^ for PEO and PEO/GO-IL (each cycle time is 4 h).

LSV has been performed to investigate the electrochemical stability of the electrolytes at 30°C and the results are shown in Figure 5A. For PEO, the oxidation stage starts at 4.0 V. In contrast, for PEO/GO-IL, there is no oxidation current until 4.48 V. The wide electrochemical window of PEO/GO-IL is very related to its modified structure. In this study, the IL-NH_2_ was grafted to GO sheets by covalent bonding, thus the agglomeration of GO can be effectively prevented and the role of GO in the composites can be well-performed. On the other hand, the addition of GO-IL filler further reduces the crystallinity of PEO. Suppression of lithium dendrite growth is crucial for the application of LMBs. Li/(PEO/GO-IL)/Li symmetrical cells were used to investigate the cycling performance and the tests were carried out under galvanostatic condition at 30°C. As shown in Figure 5B, the cells with PEO/GO-IL electrolyte can be well-charged/discharged sequentially for more than 300 h with negligible cell voltage loss at 0.1 mA cm^−2^. In contrast, for PEO, the voltage is unstable and decreases fast, and a short circuit would happened upon charging/discharging within ~150 h.

**Figure 6:**
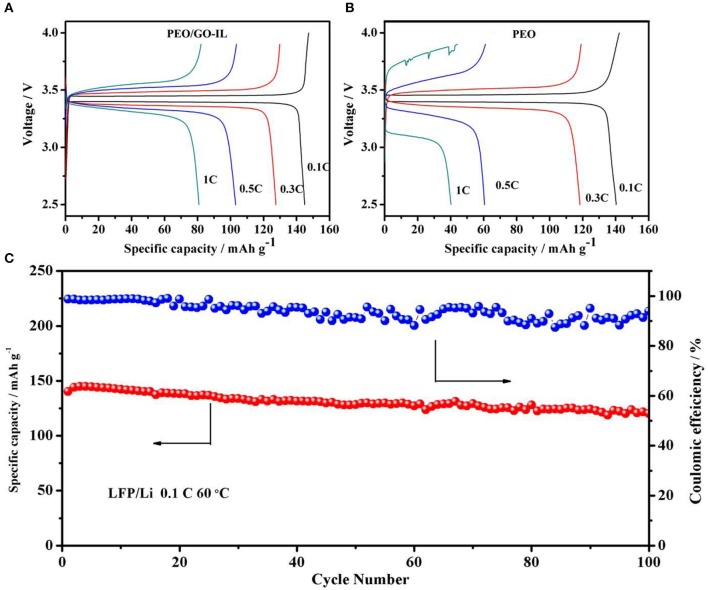
**(A)** Charge/discharge curves of cells with PEO/GO-IL at the 0.1C, 0.3C, 0.5C, 1C. **(B)** Charge/discharge curves of cells with PEO at the 0.1C,0.3C, 0.5C, 1C. **(C)** Cycling performance of cells based on PEO/GO-IL electrolyte at 0.1C at 60°C.

Solid-state LFP/Li cells with PEO and PEO/GO-IL electrolytes were assembled to verify their electrochemical performances and the electrochemical experiments were performed under different rates at 60°C. In cells using PEO/GO-IL electrolyte, polarization voltage increases slightly and the coulomb efficiency remains at 90% at 1C (Figure 6A), and the initial discharge capacity is 145 mA h g^−1^at 0.1C. In contrast, the charge/discharge curves become very rough in cells using PEO electrolyte, indicating that the internal lithium ion channel in PEO collapsed quickly when charged/discharged at 1C (Figure 6B). The great performance difference between PEO/GO-IL and PEO indicates that GO can act as a role to support the lithium ion channel in polymer.

The cycle performance of the cells with PEO/GO-IL electrolyte is evaluated at a rate of 0.1C at 60°C (Figure 6C). The cell delivers the relatively smooth charge/discharge curves and high coulombic efficiency, inferring that electrode/electrolyte interface is very compatible during charge/discharge process. Moreover, the capacity retention is as high as 88% after 100 cycles (Figure 6C), manifesting considerable electrochemical stability of PEO/GO-IL electrolyte.

## Conclusion

Novel PEO/GO-IL SPE has been developed by using GO-IL filler. The GO-IL filler is of high ionic conductivity, low crystallinity and excellent stability against the lithium anode electrode. By addition of 1% GO-IL filler, the crystallinity of PEO is significantly lowered and its performance is greatly improved. The ionic conductivity of PEO/GO-IL can reach 1.8 × 10^−5^ S cm^−1^ at 25°C and the electrolyte can effectively suppress the Li dendrite growth against Li anode electrode. Moreover, the current density for stable Li plating/stripping in the PEO/GO-IL solid electrolyte can increase to 0.1 mA cm^−2^. The investigation in this study indicated that the performance of SPEs can be greatly improved by using the fillers with excellent properties and they can effectively solve the problems of the safety and performance deterioration of Li metal anode, thus accelerating the application of LMBs.

## Data Availability Statement

The datasets generated for this study are available on request to the corresponding author.

## Author Contributions

ZH and XZ conducted the most experiments. JL and YZ performed the characterization and data analysis. All authors involved the analysis of experimental data and manuscript preparation.

### Conflict of Interest

The authors declare that the research was conducted in the absence of any commercial or financial relationships that could be construed as a potential conflict of interest.
